# Resting-State Brain Variability in Youth With Attention-Deficit/Hyperactivity Disorder

**DOI:** 10.3389/fpsyt.2022.918700

**Published:** 2022-07-12

**Authors:** Soon-Beom Hong, Seungsik Hwang

**Affiliations:** ^1^Division of Child and Adolescent Psychiatry, Department of Psychiatry, College of Medicine, Seoul National University, Seoul, South Korea; ^2^Institute of Human Behavioral Medicine, Medical Research Center, Seoul National University, Seoul, South Korea; ^3^Department of Public Health Sciences, Graduate School of Public Health, Seoul National University, Seoul, South Korea

**Keywords:** attention-deficit/hyperactivity disorder (ADHD), blood-oxygen-level-dependent (BOLD), default mode network (DMN), generalized estimating equations (GEEs), mean-square successive difference, resting-state functional magnetic resonance imaging (fMRI)

## Abstract

In this study, we sought to determine the nature of the abnormality in resting-state default mode network (DMN) activation and explore its correlation with functional connectivity in attention-deficit/hyperactivity disorder (ADHD). We obtained resting-state functional magnetic resonance images of youth with ADHD and typically developing counterparts from the publicly available ADHD-200 database. We used data from Peking University (232 scans) and New York University (172 scans); the scan repetition time was 2 s for both data collection sites. We applied generalized estimating equations to estimate the variability of the averaged blood-oxygen-level-dependent (BOLD) time series extracted from the DMN at rest. We performed network-based statistics to determine the association between the observed differences in BOLD signal variability and altered functional connectivity. We analyzed data from 105 youth with ADHD (age: mean 12.17, standard deviation 2.31, median 12.25; 15.2% female, 84.8% male) and 140 typically developing youth (age: mean 11.99, standard deviation 2.28, median 11.85; 47.1% female, 52.9% male), who aged 7–17 years. The imaging data were cross-sectionally collected for each participant at one time point. We observed a greater number of significant BOLD signal changes and higher-order polynomial significant associations in youth with ADHD. Moreover, there were significant between-group differences in BOLD signal change after the first 140 s, which coincided with decreased resting-state functional connectivity within the DMN in youth with ADHD. Increased variability of neural signaling was intermittently observed in the brains of youth with ADHD at rest, thereby indicating their default mode state was more unstable than that of typically developing youth.

## Introduction

The default mode network (DMN) is a collection of distributed brain regions that are synchronously activated during rest or internally oriented mental activities (1–3), such as mind-wandering or daydreaming (4). Introspective processes, such as thinking about self, remembering a personal past experience, or imagining a personal future event, as well as thinking about others or their mental states, commonly engage the DMN (5, 6). Contrarily, this network is deactivated while the brain performs cognitive tasks that involve external stimuli, thus exhibiting an anticorrelation with task-positive networks (7–9).

Individuals with attention-deficit/hyperactivity disorder (ADHD) have difficulties in maintaining focus on tasks and are easily distracted by unrelated thoughts (10, 11). An insufficient suppression of the DMN and the consequent intrusion of self-reflective or introspective thoughts during goal-oriented activities, or excessive spontaneous mind wandering (12), would be expected to cause attention lapses during tasks, thus interfering with effective cognitive performance (13). This, in turn, may underlie the symptoms and impairments of ADHD (14).

While unstable DMN deactivation during task engagement is characteristic of ADHD, there is limited evidence of the stability of the DMN over time during rest in this population. Despite an abundance of resting-state functional magnetic resonance imaging (rs-fMRI) studies in individuals with ADHD reporting on decreased connectivity within the DMN (15), few studies have explored its resting-state activation *per se*. Therefore, we aimed to identify abnormalities in resting-state DMN activation and determine their association with strength (i.e., an increase or decrease in activation) or stability (i.e., an increase or decrease in fluctuation). We also explored whether the nature of the abnormality is continuous (i.e., evenly distributed across time during the resting state) or intermittent (i.e., composed of intersecting periods of both normative and aberrant activations). Moreover, we aimed to compare the variability (i.e., instability and/or intermittency), key elements of DMN characteristics in individuals with ADHD, to that of typically developing counterparts. The integrity of activation of task-positive networks during a task gets interrupted by an aberrant spontaneous activation of the DMN in ADHD (12, 14). Similarly, youth with ADHD may also have trouble maintaining the integrity of DMN activation at rest. In this study, we hypothesized that intermittent abnormalities of DMN activation, rather than a sustained decrease, would more accurately capture the brains of individuals with ADHD at rest.

We further hypothesized that aberrant DMN activation may introduce greater variability in individuals with ADHD. Only a few studies have previously tested similar hypotheses. In individuals with no current or past history of psychiatric diagnosis, the blood-oxygen-level-dependent (BOLD) signal variability in rs-fMRI data decreases linearly over the lifespan (i.e., age 6–85 years) in the majority of large-scale networks. Therefore, a decrease in the resting-state signal variability may be a component of the normative trajectories of functional brain maturation (16). ADHD is considered a developmental delay in the ability to maintain focus and/or control impulses (17–19); thus, delayed maturation in the signal variability of the brain may contribute to its development. Researchers have also examined the relationship between resting-state brain signal variability and ADHD (20). Recently, the variability of neural signaling was measured in youth with and without autism spectrum disorder (ASD) (21). Both studies applied mean-square successive difference (MSSD) of the BOLD time series to measure brain signal variability (22) and found no difference in MSSD in the categorical comparison between the diagnostic and control groups; however, they observed brain-behavior associations in the dimensional analyses assessing the relationship between MSSD and symptom severity. Although a positive correlation was observed between MSSD and ADHD symptom severity in areas comprising the DMN, there was a negative correlation between MSSD and the severity of ASD behaviors in a set of distributed brain regions encompassing all four major cortical lobes. Researchers identified discrepancies in the direction of association between MSSD and symptom severity and age, thus warranting additional research.

Generalized estimating equations (GEEs) are a widely used statistical approach for modeling longitudinal data. Considering BOLD signal time series as a type of longitudinal data that are repeatedly measured at brief intervals of a few seconds, we applied GEEs to explore the changing pattern of BOLD signals in the brains of youth with and without ADHD. The *a priori* hypothesis stated that BOLD signals would be less stable or destabilized earlier in youth with ADHD. Specifically, we expected to observe a series of significant changes in BOLD signals across time. First, a greater number of significant changes will be found in youth with ADHD, thereby suggesting an increased variability of neural signaling in their brains at rest. Second, significant changes will be observed earlier in these individuals while they have trouble in maintaining the default mode state of their brains. Third, we also anticipated a greater number of higher-order (i.e., cubic > quadratic > linear) polynomial significant associations in individuals with an unstable default mode state.

In summary, we aimed to identify whether aberrant DMN activation introduces greater variability in individuals with ADHD. We expected to observe brief intermittent nature of abnormal resting-state neural signaling in youth with ADHD and their difficulty in maintaining the integrity of default mode state at rest. Accordingly, for example, no significant difference was likely to be found between the diagnostic and control groups during the first few minutes of scanning; however, the difference in BOLD signal change would be evident in a later phase of scanning, thus supporting the intermittent nature of abnormal DMN activation in individuals with ADHD. After exploring the variability of resting-state BOLD signal changes, we further explored the correlation between the observed instability of DMN activation and decreased functional connectivity within the DMN of youth with ADHD (15).

## Materials and Methods

### Participants and Data Acquisition

We obtained the study data from the publicly available ADHD-200 database (http://fcon_1000.projects.nitrc.org/indi/adhd200/) and ADHD-200 Preprocessed repository (http://preprocessed-connectomes-project.org/adhd200/). The complete dataset consisted of MRI scans and phenotypic information collected from eight international sites. To ameliorate concerns about different scanners and scanning parameters among the collection sites, we aimed to reduce the number of data collection sites but retain maximum participants. Considering our purpose to detect instability and/or intermittency of BOLD time series, we prioritized rs-fMRI data with greater measurements (i.e., number of scans), in addition to a larger sample size. Consequently, we used data from Peking University (232 scans) and New York University (172 scans), the two largest data collection sites in the ADHD-200 dataset. For participants whose brain images were collected more than once, we used images from the first data collection.

The presence of ADHD was based on evaluations using the Schedule of Affective Disorders and Schizophrenia for Children–Present and Lifetime Version at both sites. Right-handedness and a full-scale intelligence quotient (IQ) above 80 were required to participate. The severity of ADHD symptoms were assessed using the Attention-Deficit/Hyperactivity Disorder Rating Scale-IV (ADHD-RS-IV) and the Conners' Parent Rating Scale-Revised, Long version (CPRS-R-LV) at Peking University and New York University, respectively. Participants withheld psychostimulants at least 48 and 24 h before scanning at Peking University and New York University, respectively.

The ADHD-200 data include in-house quality assessment results for both structural MRI and rs-fMRI scans, which are binary data of either “pass” or “questionable.” We included youth with “pass” for both structural MRI and rs-fMRI scans and no missing IQ scores. Head motion during scanning is another critical concern in rs-fMRI studies. Based on the 10 head motion parameters provided in the ADHD-200 data, we labeled values over 2.2 interquartile range units below the lower or above the upper quartile as outliers (23). We excluded youth with at least one outlier among the 10 head motion parameters. Specifically, among the 194 participants from Peking University and 216 from New York University whose data were preprocessed and released based on the Athena pipeline (24), 178 and 111 participants were retained, respectively, based on the “pass” image quality. After excluding those with the outliers (see Supplemental Table 1 for details), 153 and 101 participants were eligible from the two sites, respectively. Among these, one and eight participants were further excluded for missing IQ scores, respectively. Ultimately, we analyzed data from 105 youth with ADHD (age: mean 12.17, standard deviation 2.31, median 12.25; 15.2% female, 84.8% male) and 140 typically developing youth (age: mean 11.99, standard deviation 2.28, median 11.85; 47.1% female, 52.9% male), who aged 7–17 years.

### Image Processing

We used the preprocessed rs-fMRI data released by the Neuro Bureau ADHD-200 Preprocessed repository. Images were preprocessed using the Athena pipeline (24). We obtained regionally averaged BOLD signals across voxels from the DMN regions using Data Processing & Analysis of Brain Imaging (25). We used Yeo's 7-resting-state network parcellation atlas (26), which was resampled to the space of functional images.

### Data Analysis

We estimated between-group differences in descriptive statistics and head motion parameters using Student's *t*-tests and chi-square tests for continuous and categorical variables, respectively. We applied GEEs to the averaged BOLD time series from the DMN. Due to the correlated structure of data (i.e., BOLD signals) from repeated measurements, we used GEE, which assumes neither a normal distribution nor independent data. The scan repetition time was 2 s for both data collection sites. To match the number of measurements, we discarded some of the later scans from Peking University and analyzed 172 scans for both New York University and Peking University. To achieve our study objectives, we carried out two sets of GEE analyses, with three models in each set of analyses. Considering numerous measurements, we did not expect the entire time series to fit a specific (e.g., linear, quadratic, and cubic) model; thus, we split the entire time series into small units of five scans (or 10 s) each and subsequently applied a GEE to each unit.

First, we separately analyzed youth with ADHD and healthy controls to explore changes in the averaged BOLD signals from the DMN across time in each group. We tested three models, namely, linear, quadratic, and cubic, where we consecutively added the z-transformed time (zTime), zTime squared, and zTime cubed to the models, along with sex and age. The time variable was the sequential order of the scanning time points, ranging from 1 to 172. The dependent variable was the resting-state BOLD signal.

Second, statistically significant differences in BOLD signal change between the ADHD and control groups were tested by combining the groups and entering the interaction terms between diagnosis and time variables. The GEE model 1 included age, sex, diagnosis, zTime, and the interaction of diagnosis × zTime, whereas we added zTime squared and the interaction of diagnosis × zTime squared in model 2, and zTime cubed and the interaction of diagnosis × zTime cubed in model 3. The dependent variable was the resting-state BOLD signal.

We also tested an alternative measure of signal variability, the MSSD (16, 20, 21, 27, 28). The MSSD of the BOLD time series was calculated and compared between the ADHD and control groups using Student's *t*-tests. Using Pearson's correlation coefficient, we separately determined the association between ADHD symptom severity and MSSD for Peking University and New York University samples. This is because different measures of ADHD symptoms were adopted by the two data collection sites. We performed statistical tests, including the GEE analyses, using SPSS (version 25.0; IBM, Armonk, NY, USA), and reported the results with a significance threshold of uncorrected *P* < 0.05. In addition, since the entire time series were split into small units and the tests were repeated for each unit, we applied a false discovery rate (FDR) correction for multiple comparisons.

Third, we tested an association between the instability in BOLD signals in the resting-state DMN and altered functional connectivity. We extracted BOLD signals from 91 DMN regions of interest of Schaefer's 7-network 400-region cortical parcellation (29) and performed network-based statistics (NBS) to separately identify significant between-group differences in the inter-regional functional connectivity for the first 70 scans and the next 70 scans (30, 31). The 70-scan criterion was based on the results of preceding GEE analyses. We grouped connections that survived the *a priori* cluster-defining threshold of three and shared nodes in common into a cluster of suprathreshold edges. Subsequently, we calculated family-wise error (FWE) probabilities for the number of connections in the cluster through 10,000 permutation tests. We included sex, IQ, and one (i.e., maximum rotation time point) of the 10 head motion parameters as covariates owing to their significant difference between the groups. Age was not included as a covariate, given the absence of a between-group difference. We performed these steps using the NBS software package (http://www.nitrc.org/projects/nbs/) and visualized the results using BrainNet Viewer (http://www.nitrc.org/projects/bnv/) (32).

## Results

### Participant Characteristics

There were no significant differences in age and data collection sites between youth with ADHD and controls (Table 1). The groups significantly differed in terms of sex, IQ, and ADHD symptom severity scores. The ADHD group showed a higher proportion of male participants and a lower IQ score than controls. Of the 10 head motion parameters, the maximum rotation time point was significantly different between the groups which was increased in the control group.

**Table 1:** Characteristics of study participants.

	**ADHD (*n* = 105)**		**Control (*n* = 140)**		*P*-value
Age (years), Mean (SD)	12.17	2.31	11.99	2.28	0.532
Sex (female), *n* (%)	16	15.2	66	47.1	<0.001
IQ, Mean (SD)	105.45	13.59	115.53	14.04	<0.001
ADHD-RS (Peking), Mean (SD)					
Total score	49.90	8.55	28.24	6.15	<0.001
Inattention score	27.95	3.41	14.97	3.67	<0.001
Hyperactivity/impulsivity score	21.95	6.52	13.27	3.56	<0.001
CPRS-R (New York), Mean (SD)					
ADHD index score	71.21	9.34	45.22	4.53	<0.001
Inattention score	70.83	10.05	45.02	4.60	<0.001
Hyperactivity score	67.43	12.46	46.22	5.02	<0.001
Data collection site, *n* (%)					0.569
Peking	63	60.0	89	63.6	
New York	42	40.0	51	36.4	
Head motion parameters, Mean (SD)					
Maximum motion (mm)	0.84	0.57	0.76	0.51	0.235
Maximum motion (time point)	171.52	52.32	182.84	41.70	0.061
Maximum rotation (degree)	0.81	0.56	0.69	0.46	0.076
Maximum rotation (time point)	157.02	59.64	172.85	51.51	0.027
Maximum translation, x-axis (mm)	0.04	0.19	0.05	0.17	0.536
Maximum translation, y-axis (mm)	−0.20	0.36	−0.28	0.38	0.116
Maximum translation, z-axis (mm)	0.00	0.81	−0.15	0.70	0.106
Maximum roll rotation (degree)	−0.05	0.30	−0.07	0.25	0.471
Maximum pitch rotation (degree)	0.11	0.80	0.13	0.68	0.879
Maximum yaw rotation (degree)	0.04	0.35	0.10	0.28	0.139

*No missing observations, except for the ADHD indices. ADHD-RS scores were available for 58 patients with ADHD and 77 control participants. The CPRS-R scores were available for 42 patients with ADHD and 50 control participants*.

*ADHD, attention-deficit/hyperactivity disorder; ADHD-RS, ADHD Rating Scale; CPRS-R, Conners' Parent Rating Scale-Revised; IQ, intelligence quotient; and SD, standard deviation*.

### Within-Group BOLD Signal Changes

**Figure 1:**
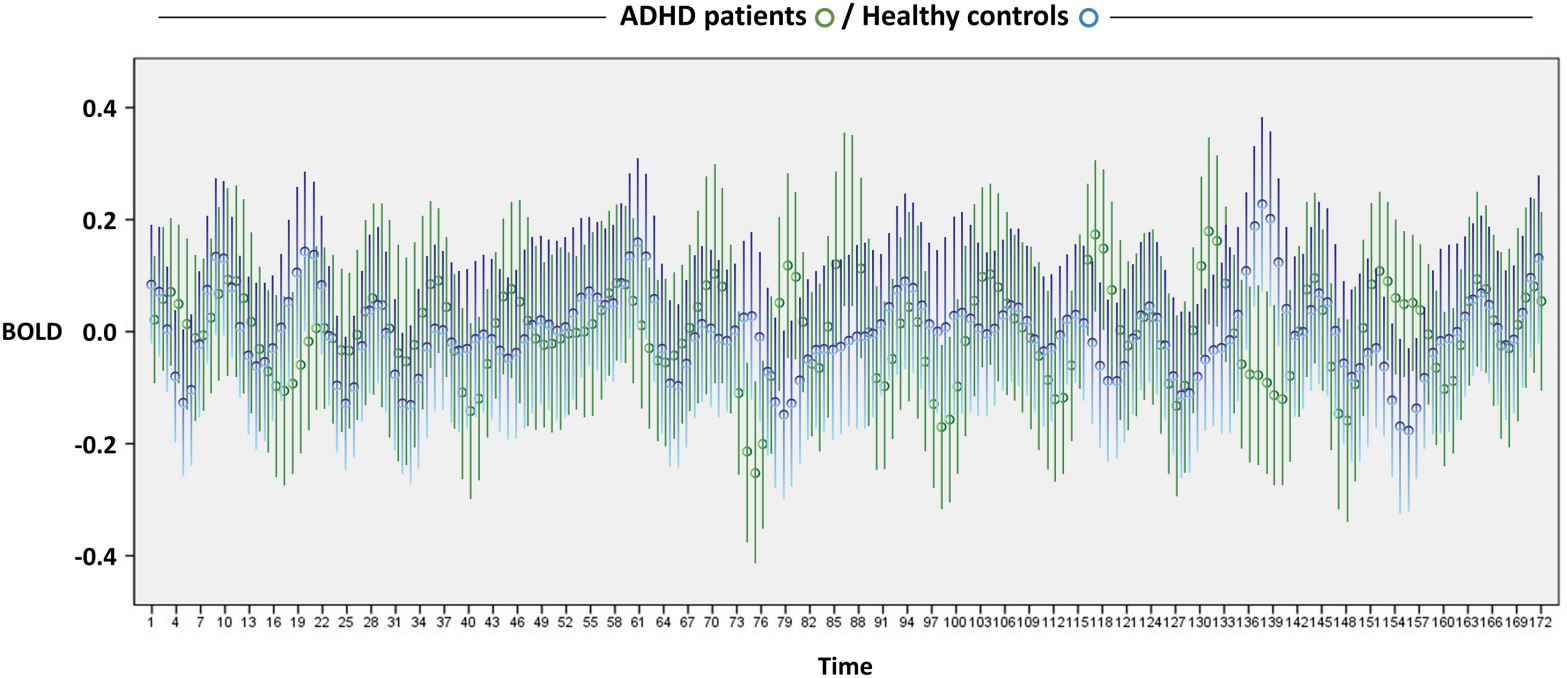
Resting-state BOLD fluctuation across 5 min and 44 s. Circles and bars indicate the mean and 95% confidence intervals, respectively. ADHD, attention-deficit/hyperactivity disorder; BOLD, blood-oxygen-level-dependent.

**Figure 2:**
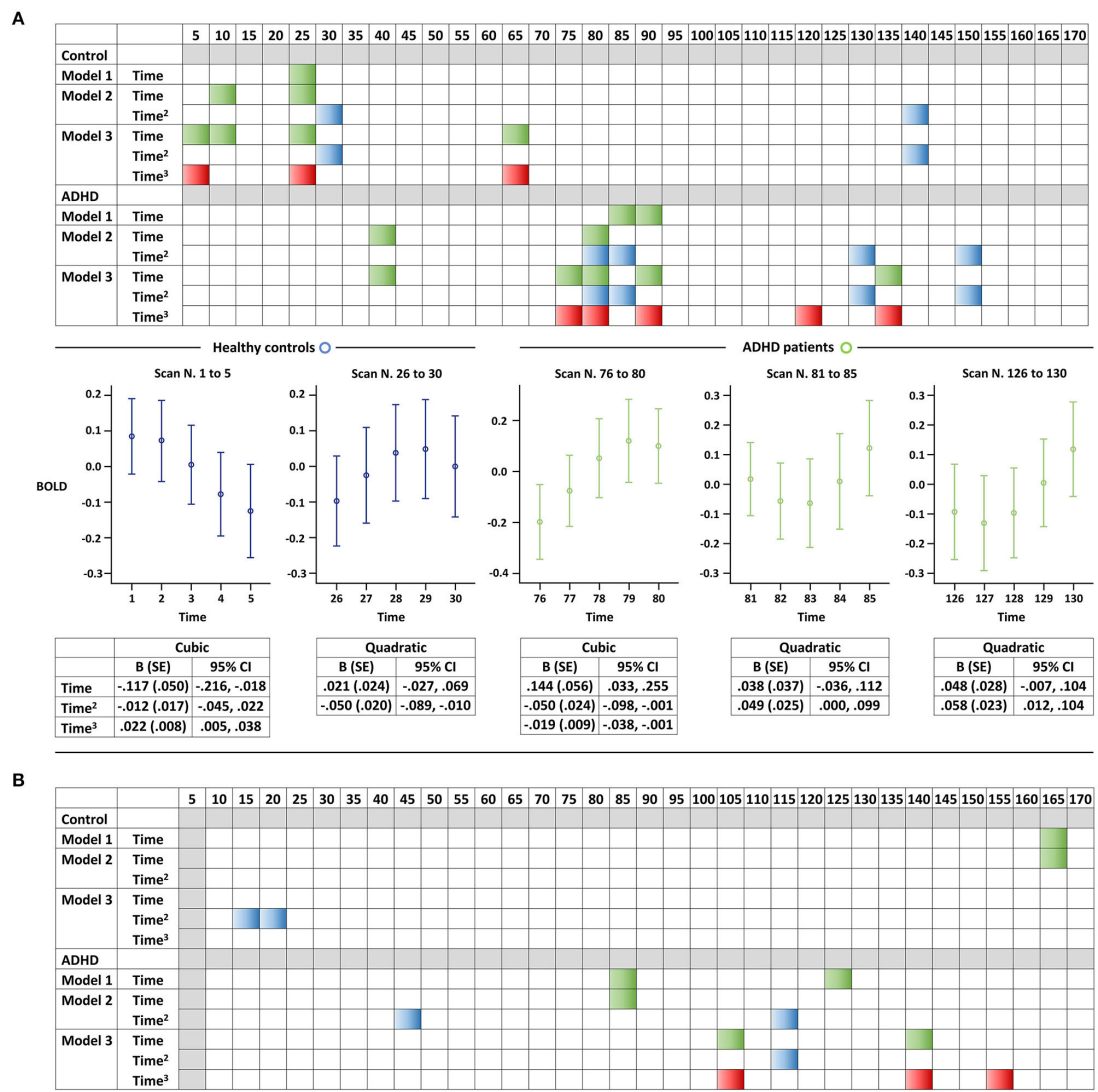
Resting-state BOLD signal changes in the default mode network of youth, with and without attention-deficit/hyperactivity disorder. Representation of the results of the GEE analyses for each **(A)** five-scan and **(B)** 10-scan unit. We displayed each of the three models separately (e.g., linear, quadratic, and cubic) considering the exploratory nature of the study, and a selection of graphs is presented as examples to help readers better understand the colored panels. Significant associations of a linear, quadratic, or cubic term for z-transformed time with BOLD signals (*P* < 0.05) are illustrated in green, blue, and red, respectively. Error bars illustrate the five-scan BOLD signals along with detailed statistics of the selected models. ADHD, attention-deficit/hyperactivity disorder; BOLD, blood-oxygen-level-dependent; and GEE, generalized estimating equation.

Figure 1 depicts the BOLD signal fluctuation for the entire time series in the DMN of youths with and without ADHD. In the separate GEE analysis for youth with ADHD and controls, we observed a greater number of significant BOLD signal changes and of higher-order (i.e., cubic > quadratic > linear) polynomial significant associations in the patients, as expected *a priori* (Figure 2A). We replicated these findings on changing the unit of analysis from 5 to 10 scans (Figure 2B). However, there were unexpected significant BOLD signal changes in the earliest scans (i.e., within the first 1 min) in the control group, particularly for five scans. This was not aligned with our expectation that significant BOLD signal changes would be evident in a later phase of scanning. Overall, based on simple counting, the control youth exhibited marginal signal change throughout scanning, while those with ADHD displayed greater signal changes, particularly in the latter part of scanning (Figure 2). No significant BOLD signal changes were observed based on the FDR correction for multiple comparisons, regardless of whether the unit of analysis was 5 or 10 scans.

### Between-Group Differences in BOLD Signal Change

**Figure 3:**
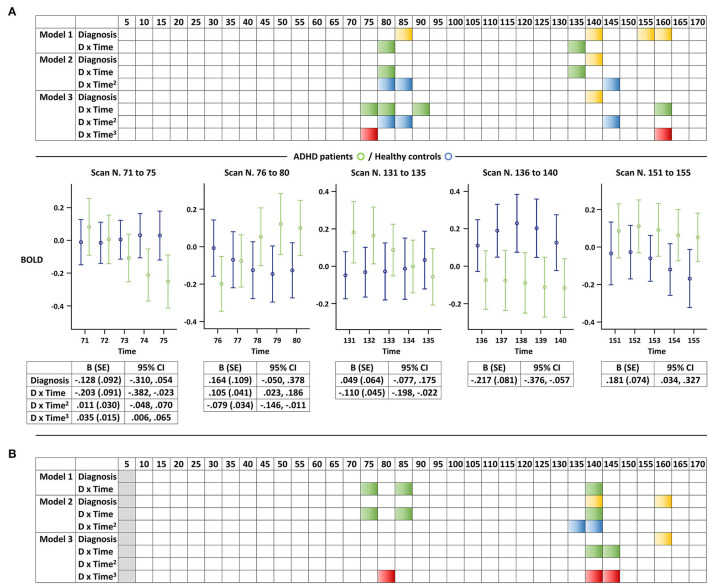
Altered resting-state BOLD signal changes in the default mode network of youth with attention-deficit/hyperactivity disorder. Representation of the results of the GEE analyses for each **(A)** five-scan and **(B)** 10-scan unit. We displayed each of the three models separately (e.g., linear, quadratic, and cubic) considering the exploratory nature of the study, and a selection of graphs is presented as examples to help readers better understand the colored panels. Significant associations of an interaction term between diagnosis and *z*-transformed time, *z*-transformed time squared, or *z*-transformed time cubed with BOLD signals (*P* < 0.05) are illustrated in green, blue, and red, respectively. Significant associations between diagnosis and BOLD signals (*P* < 0.05) are illustrated in yellow. Error bars illustrate the five-scan BOLD signals along with detailed statistics of the selected models. ADHD, attention-deficit/hyperactivity disorder; BOLD, blood-oxygen-level-dependent; and GEE, generalized estimating equation.

In the combined GEE analysis for youth with ADHD and controls, changes observed in the control group in the earliest scans did not introduce significant between-group differences (Figure 3). In addition, BOLD signal changes significantly differed only in the latter part of scanning, especially after the first 70 scans (or 140 s), thus supporting the intermittent nature of abnormal DMN activation in youth with ADHD. These findings were comparable when the unit of analysis was either 5 or 10 scans. No significant between-group differences in BOLD signal changes were observed based on the FDR correction for multiple comparisons, regardless of whether the unit of analysis was 5 or 10 scans.

### Additional Analyses Using MSSD

There was no significant difference in MSSD between youth with ADHD and controls. No significant correlation was observed between MSSD and ADHD symptom severity (i.e., ADHD-RS-IV total, inattention, and hyperactivity/impulsivity scores from Peking University and CPRS-R-LV ADHD index, inattention, and hyperactivity scores from New York University) in youth with ADHD as well as in typically developing controls, regardless of MSSD calculated for the entire time series, the first 70 scans, or the next 70 scans. When the Pearson correlation analyses were performed separately among female and male participants, no significant correlations were observed.

### Relationships Between BOLD Signal Variability and Connectivity

**Figure 4:**
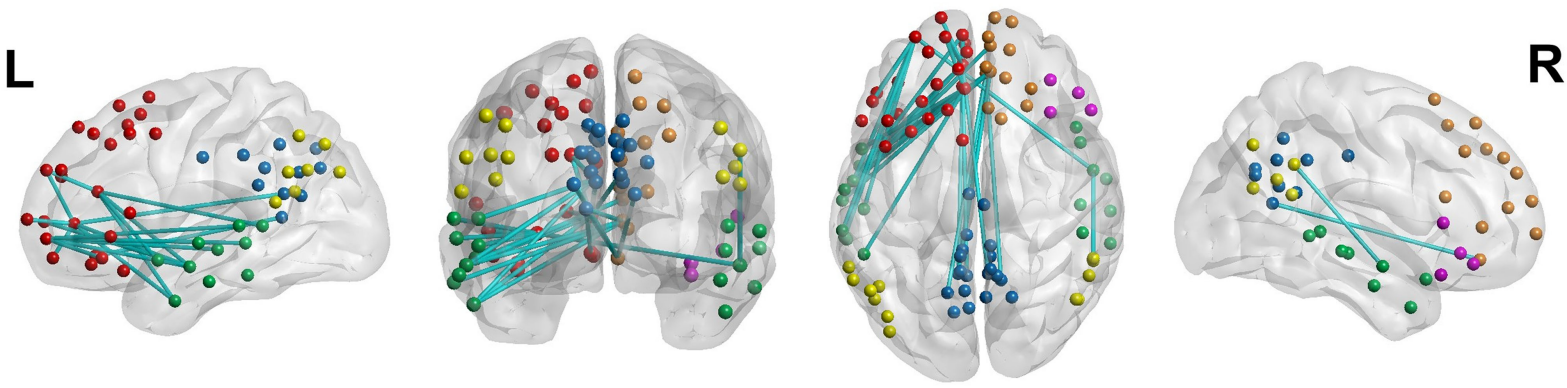
Decreased functional connectivity in the default mode network of youth with attention-deficit/hyperactivity disorder. Nodal colors are presented in green, yellow, red, blue, pink, and orange in the temporal cortex, parietal cortex, prefrontal cortex, precuneus and posterior cingulate cortex, ventral prefrontal cortex, and dorsal and medial prefrontal cortex, respectively, all within the default-mode network.

Considering the significant differences identified from the GEE analyses after the first 70 scans, we separately applied NBS to the first 70 scans and the next 70 scans, thereby predicting a significant difference in the functional network only in the next set of scans. The NBS analysis did not reveal significant between-group differences in the first 70 scans, whereas a network with significantly decreased connectivity was observed within the DMN of youth with ADHD using the next 70 scans (FWE-corrected *P* < 0.05, 10,000 permutations; Figure 4, Supplemental Table 2).

## Discussion

We observed intermittent changes in resting-state BOLD signal fluctuation in youth with and without ADHD. As expected *a priori*, a greater number of significant changes and of higher-order (i.e., cubic > quadratic > linear) polynomial significant associations were observed in youth with ADHD, thus suggesting the possibility of increased moment-to-moment BOLD variability in their DMN at rest. In addition, we identified significantly abnormal resting-state DMN activation in these individuals. As predicted *a priori*, the nature of the aforementioned abnormality was intermittent rather than continuous. Moreover, significant differences were principally observed in the interactions between diagnosis and time variables rather than the diagnosis *per se*, thereby suggesting the core feature of the abnormality lies in the variability of BOLD fluctuation over time, and not in the strength of BOLD activation. It is important to note that the between-group differences were not observed in the overall analyses, but only in the later scans, indicating a certain degree of abnormality in the maintenance of the resting-state DMN activation. In summary, increased variability of neural signaling was observed in the brains of youth with ADHD at rest, which indicated their default mode state was more unstable than that of typically developing youth.

Changes in the earliest scans in the control group were unexpected and indicated a supposedly more rapid BOLD stabilization in youth with ADHD at the beginning of rest, despite no between-group difference in the combined analysis. It is unclear if this finding reflected another core feature of ADHD (i.e., a more rapid default mode engagement) or the need to discard more initial scans before the analysis. The preprocessing of the Athena pipeline removed a relatively small number of the first four volumes, compared with other studies.

We also observed decreased resting-state functional connectivity within the DMN in youth with ADHD, consistent with previous findings (15). However, this study added to the literature that decreased connectivity might not be uniformly distributed across the resting state. Instead, it was observed only in the later scans, coinciding with the period when the variability of neural signaling increased. In other words, the DMN of youth with ADHD appeared to function similarly to that of the typically developing youth for a relatively short duration; however, youth with ADHD might have difficulties in maintaining it.

Across the 91 DMN regions of interest included in the study, 28 regions were involved in the network with significantly decreased connectivity in youth with ADHD. Resting-state functional connectivity is measured based on the correlations of brain activity across different brain areas and may thus be closely related to the moment-to-moment changing pattern of neural signaling in each of these areas. Further work is required in order to better understand the relationship between BOLD signal variability and functional connectivity.

In this study, we examined a novel approach for exploring moment-to-moment BOLD signal changes. The GEE is far from being novel; however, it has been rarely applied in neuroimaging research. Contrarily, MSSD is one of the most commonly used measures of resting-state brain signal variability and has been investigated in ADHD (20), ASD (21), and schizophrenia (28). None of these studies observed significant differences in MSSD in the DMN of the brain, besides inconsistencies persisting in the direction of associations between MSSD and symptom severity. In line with previous studies, we did not observe a significant difference in MSSD between the ADHD and control groups. MSSD can be a powerful measure of variability in cases with frequent aberrant variability throughout the entire time series. In contrast, it can be less sensitive in detecting intermittent abnormalities of relatively short durations embedded in the middle of lengthy normative data.

This study had some limitations. First, youth with and without ADHD were not strictly matched for sex and IQ. Considering our primary purpose was to separately explore the variability of BOLD fluctuation in youth with ADHD and typically developing youth, we did not perform case-by-case matching, which would reduce the sample size. Instead, we opted to maximize our sample size in each group as well as maintain the typical characteristics of the ADHD population (33), such as the predominance of male participants (34) and a loss of approximately nine points in the IQ score (35). The exclusion of youth with ADHD and relatively lower IQ would result in the ADHD group inaccurately representing the actual population (36, 37). Furthermore, if ADHD and IQ were negatively correlated, controlling for the IQ as a covariate while examining group differences in cognitive functioning would eliminate some ADHD-mediated variance (36, 37). Based on these concerns, we included sex but not IQ as a covariate in the GEE analyses. Another reason was that the first set of GEE analyses were carried out separately in youth with and without ADHD, mitigating the concern on IQ difference. We then maintained the covariates in the following set of between-group analyses for consistency. However, these findings need to be replicated in a different dataset better matched for sex, as well as IQ. Likewise, lying motionless in the scanner can be more challenging for individuals with ADHD, which can be regarded as part of their typical characteristics. However, head motion in the scanner can have serious confounding influences in rs-fMRI studies (38, 39), which would be problematic even in the separate analyses for each group; therefore, we adopted the independent criteria for identifying outliers to exclude those who moved more than expected regardless of their diagnostic status. Nevertheless, it remains possible that the first 70 and the next 70 scans had different image quality due to relatively increased head motion during the latter. This may have influenced the analyses. However, it would be very difficult to completely eliminate this influence, and perhaps questions would be raised with regard to whether it should be removed, especially in the study of ADHD, since difficulty in lying motionless over an extended time would be a typical characteristic of children with ADHD, if not that of children (or even adults) in general. In this study, however, children with ADHD did not appear to be particularly vulnerable to motion in the scanner, considering that the only difference in motion parameters was an increase in maximum rotation in the control group. Of note in this study, the number of participants excluded from the analysis due to outliers in maximum rotation was significantly greater in the ADHD group (see Supplemental Table 1). Second, we used data from two different sites, namely, Peking University and New York University, raising the concern of potential bias from differences in MRI machines. However, no difference was found between the two sites in the proportions of ADHD and control participants, which were relatively well-matched with a ratio of approximately 3:2 at both sites; thus, it may have been less likely to introduce spurious between-group differences. Third, we used Yeo's 7-resting state network parcellation atlas and the entire DMN region of interest. The DMN is a collection of distributed brain regions, and we did not test our hypothesis in each of these regions separately. Although these regions are synchronously activated during rest, their activities may show some inconsistencies. Therefore, if we had tested our hypothesis in each of these regions separately, some of them would likely coincide with our hypothesis, whereas others would not. Hence, we intended to focus on the synchronous component of the signals by targeting the entire DMN as a whole and analyzing the regionally averaged BOLD signals. Nevertheless, further work is needed to clarify whether the findings are robust with other atlases or in different subregions of the network. Fourth, as a resting-state study, there was no time-locked driver for BOLD signals similar to that in task-based fMRI, which can synchronize the temporal event more effectively across the participants. For example, the start time of each participant's resting mental state may have been shortly after they were lying down in the scanner, but if the start time of the actual scanning had been a variable (e.g., delayed for some participants), it may have introduced confounding influences. However, no data were collected on the duration between the beginning of a participant's resting mental state and that of their fMRI data acquisition. Further studies are warranted to verify whether these findings are a reliable pathological effect or some coincidence in the fluctuating resting-state BOLD signals. Fifth, the findings were not significant after correction for multiple comparisons. However, it may be noteworthy that we did not aim to test a null hypothesis (e.g., BOLD signals do not change across time points) but to explore the nature of signal change across different time points and different participants. Calculating MSSD for all time points may not be sufficiently sensitive to detect brief intermittent changes in variability, whereas dividing them into small units of short duration and applying GEE to each unit can be more sensitive. However, it may introduce the problem of multiple testing. This warrants more sophisticated statistical methods for estimating the variability and/or intermittency of BOLD fluctuations.

In conclusion, this study indicated that the variability in resting-state BOLD fluctuation increased in the DMN of youth with ADHD, particularly in the later phase of scanning. Increased variability was intermittently observed and related to decreased functional connectivity within the DMN, compared with typically developing youth. To the best of our knowledge, this is the first study to apply GEE to investigate the changing patterns of resting-state BOLD signals. Further studies are required on the variable and intermittent nature of aberrant resting-state neural signaling in individuals with ADHD and their difficulty in maintaining the default-mode state.

## Data Availability Statement

The data used in this study are publicly available from the ADHD-200 database (http://fcon_1000.projects.nitrc.org/indi/adhd200/) and Preprocessed repository (http://preprocessed-connectomes-project.org/adhd200/).

## Ethics Statement

This study used deidentified data acquired from an open data repository and was granted exemption from review by the Institutional Review Board at the Seoul National University Hospital.

## Author Contributions

S-BH was responsible for the study concept and design, downloaded and analyzed the data, and drafted the manuscript. SH assisted with data analysis and interpretation of findings, and provided critical revision of the manuscript for important intellectual content. Both authors critically reviewed content and approved final version for publication.

## Conflict of Interest

The authors declare that the research was conducted in the absence of any commercial or financial relationships that could be construed as a potential conflict of interest.

## Publisher's Note

All claims expressed in this article are solely those of the authors and do not necessarily represent those of their affiliated organizations, or those of the publisher, the editors and the reviewers. Any product that may be evaluated in this article, or claim that may be made by its manufacturer, is not guaranteed or endorsed by the publisher.
