# Supplementary material for: Resting-State Brain Variability in Youth With Attention-Deficit/Hyperactivity Disorder
Source: Front Psychiatry. 2022 Jul 12;13:918700. doi: 10.3389/fpsyt.2022.918700 (PMC9322108; doi:10.3389/fpsyt.2022.918700)
Supplement: Supplementary file 1 [file Data_Sheet_1.docx]

Supplemental Table 1. Number of participants with outliers in each head motion parameter

|  | **Peking University** | | | **New York University** | | |
| --- | --- | --- | --- | --- | --- | --- |
|  | **ADHD** | **Control** | **p-value** | **ADHD** | **Control** | **p-value** |
| Maximum motion, *n* (%) | 2 (2.8) | 3 (2.8) | 0.983 | 0 (0.0) | 2 (3.3) | 0.196 |
| Maximum motion, *n* (%) | 2 (2.8) | 2 (1.9) | 0.694 | 0 (0.0) | 0 (0.0) | N/A |
| Maximum rotation, *n* (%) | 3 (4.2) | 4 (3.8) | 0.895 | 4 (8.0) | 0 (0.0) | 0.024 |
| Maximum rotation, *n* (%) | 0 (0.0) | 0 (0.0) | N/A | 0 (0.0) | 0 (0.0) | N/A |
| Maximum translation, x-axis, *n* (%) | 3 (4.2) | 5 (4.7) | 0.862 | 1 (2.0) | 1 (1.6) | 0.887 |
| Maximum translation, y-axis, *n* (%) | 1 (1.4) | 4 (3.8) | 0.345 | 1 (2.0) | 2 (3.3) | 0.679 |
| Maximum translation, z-axis, *n* (%) | 3 (4.2) | 4 (3.8) | 0.895 | 0 (0.0) | 2 (3.3) | 0.196 |
| Maximum roll rotation, *n* (%) | 1 (1.4) | 1 (0.9) | 0.782 | 0 (0.0) | 2 (3.3) | 0.196 |
| Maximum pitch rotation, *n* (%) | 2 (2.8) | 4 (3.8) | 0.718 | 4 (8.0) | 0 (0.0) | 0.024 |
| Maximum yaw rotation, *n* (%) | 2 (2.8) | 4 (3.8) | 0.718 | 0 (0.0) | 1 (1.6) | 0.363 |

Values over 2.2 interquartile range units below the lower or above the upper quartile were labeled as outliers. The between-group differences in the number of participants with outliers were estimated using chi-square tests.

ADHD, attention-deficit/hyperactivity disorder

Supplemental Table 2. Brain regions involved in the default mode network with significantly decreased connectivity in youth with ADHD

| **Hemisphere** | **Component** |  | **Hemisphere** | **Component** |
| --- | --- | --- | --- | --- |
| Left | Prefrontal 6 | ↔ | Left | Temporal 4 |
| Left | Prefrontal 6 | ↔ | Left | Temporal 6 |
| Left | Prefrontal 6 | ↔ | Left | Temporal 7 |
| Left | Prefrontal 6 | ↔ | Left | Temporal 9 |
| Left | Prefrontal 6 | ↔ | Left | Prefrontal 5 |
| Left | Prefrontal 7 | ↔ | Left | Prefrontal 6 |
| Left | Prefrontal 8 | ↔ | Left | Temporal 4 |
| Left | Prefrontal 9 | ↔ | Left | Temporal 1 |
| Left | Prefrontal 9 | ↔ | Left | Temporal 4 |
| Left | Prefrontal 9 | ↔ | Left | Prefrontal 3 |
| Left | Prefrontal 9 | ↔ | Left | Prefrontal 8 |
| Left | Prefrontal 11 | ↔ | Left | Prefrontal 9 |
| Left | Prefrontal 12 | ↔ | Left | Temporal 1 |
| Left | Prefrontal 12 | ↔ | Left | Temporal 4 |
| Left | Prefrontal 12 | ↔ | Left | Temporal 5 |
| Left | Prefrontal 12 | ↔ | Left | Temporal 10 |
| Left | Prefrontal 14 | ↔ | Left | Prefrontal 9 |
| Left | Prefrontal 14 | ↔ | Left | Prefrontal 12 |
| Left | Precuneus & posterior cingulate 1 | ↔ | Left | Prefrontal 4 |
| Left | Precuneus & posterior cingulate 3 | ↔ | Left | Prefrontal 9 |
| Right | Temporal 4 | ↔ | Left | Prefrontal 6 |
| Right | Temporal 4 | ↔ | Right | Parietal 4 |
| Right | Dorsal & medial prefrontal 1 | ↔ | Left | Precuneus & posterior cingulate 1 |
| Right | Dorsal & medial prefrontal 1 | ↔ | Left | Precuneus & posterior cingulate 2 |
| Right | Dorsal & medial prefrontal 3 | ↔ | Left | Temporal 1 |
| Right | Dorsal & medial prefrontal 3 | ↔ | Left | Temporal 2 |
| Right | Dorsal & medial prefrontal 3 | ↔ | Left | Temporal 4 |
| Right | Dorsal & medial prefrontal 3 | ↔ | Left | Precuneus & posterior cingulate 1 |
| Right | Dorsal & medial prefrontal 4 | ↔ | Left | Temporal 4 |
| Right | Precuneus & posterior cingulate 1 | ↔ | Right | Dorsal & medial prefrontal 1 |
| Right | Precuneus & posterior cingulate 4 | ↔ | Left | Prefrontal 9 |

The network consisted of 31 links involving 28 different brain regions.

ADHD, attention-deficit/hyperactivity disorder
